# Impact of weight-bearing on foot shape: a geometric and morphometric assessment using principal polynomial shape analysis

**DOI:** 10.1038/s41598-026-51650-4

**Published:** 2026-05-12

**Authors:** Jing Li, Cédric Bonte, Emmanuel Audenaert, Arne Burssens, Matthias Peiffer, Roel Huysentruyt, Ide Van den Borre, Aline Van Oevelen, Kate Duquesne

**Affiliations:** 1 Department of Human Structure and Repair, Ghent University, Corneel Heymanslaan 10, 9000 Gent, OVL Belgium; 2 Department of Orthopedic Surgery and Traumatology, Ghent University Hospital, Ghent, Belgium

**Keywords:** Weight-bearing computed tomography (WBCT), Foot, Image analysis, Shape modelling, Anatomy, Engineering, Health care, Medical research

## Abstract

Weight-bearing computed tomography (WBCT) has set a new standard for assessing foot alignment under physiological loading conditions. However, few studies have systematically quantified morphological differences between weight-bearing (WB) and non-weight-bearing (NWB) conditions. Therefore, the purpose of this study was to evaluate WB-induced foot shape changes using Principal Polynomial Shape Analysis (PPSA), a recent non-linear technique in geometric morphometrics. In this retrospective matched-pair study, we analysed 78 feet from 39 subjects scanned under WB conditions (WBCT-based) and 78 feet from 39 matched subjects scanned under NWB conditions (MRI-based). 3D foot bone models were reconstructed, registered, and compared. Radiographic measurements were calculated and statistically analysed between groups. As a result, PPSA captured 65.4% and 69.6% of shape variance in the first three components for the WB and NWB groups, respectively. Distance mapping revealed morphological deviations of up to 13 mm between the two groups, with the most statistically significant differences localised at the talonavicular joint (P < 0.05). These findings were further supported by radiographic measurements (P < 0.05). This study demonstrates that PPSA effectively captured subtle yet systematic shape changes induced by WB, offering a more comprehensive representation of WB–NWB morphological differences than conventional 2D radiographic measurements.

## Introduction

By interconnecting bones and soft tissue structures, the human foot forms a complex three-dimensional (3D) framework essential for stability, flexibility and shock absorption during weight-bearing (WB) activities such as standing, walking, and running^1^. However, the complex structure of the foot makes it susceptible to structural deformities and functional impairments under abnormal mechanical loading^2^. Clinically, foot pathologies pose diagnostic challenges due to the anatomical diversity of the forefoot, midfoot, and hindfoot regions, which often present with overlapping symptoms and subtle structural malalignments, particularly during the early stages of disease development^3^.

Traditional diagnostic imaging methods, such as WB radiographs and conventional computed tomography (CT), exhibit notable limitations. Radiographs provide two-dimensional (2D) projections in which anatomical structures are prone to superimposition, whereas standard CT produces 3D reconstructions but is typically acquired in a non-weightbearing (NWB) position, thereby lacking the ability to assess functional anatomy under physiological loading conditions^4^. These limitations highlight the necessity for advanced imaging modalities to capture foot morphology under physiological WB conditions^5^. Weight-bearing computed tomography (WBCT) addresses this need by providing precise 3D visualisation of skeletal structures and joint alignment under WB conditions^6,7^. As a relatively recent innovation, WBCT significantly enhances diagnostic accuracy for complex foot disorders^5,6,8^. Although WBCT has substantially advanced the 3D assessment of complex foot disorders, many existing studies still rely predominantly on 2D radiographic radiographic measurements—such as angles, rotations, and inter-bone distances—rather than holistic, shape-based analyses^3,9,10^. Such 2D radiographic measures reduce the inherently complex 3D morphology of the foot to a limited set of scalar descriptors, leading to a loss of potentially important geometric information that is distributed across the full 3D structure^10,11^.

Alternatively, geometric and morphometrics offer robust and precise means for characterising the complete anatomical shape and its variability across populations^12,13^. These methods extract quantitative information on shape by analysing sets of homologous landmarks, enabling conventional multivariate statistical analyses to describe anatomical variation within populations^13,14^. Unlike previous methods, these data-driven techniques offer precise parameterisations of individual morphologies, opening up new avenues for investigating anatomical distributions and their implications for medical diagnosis, classification, and treatment^13^. Within this framework, principal component analysis (PCA)-based statistical shape modelling (SSM) has become the most widely adopted approach^15,16^. Although PCA-based SSMs can capture arch deformation and rotational variations, these changes are typically represented through linear statistical modes^10,17^. Such linear dependence may limit the ability of these models to describe more complex or nonlinear anatomical patterns^17^. To address this limitation, principal polynomial shape analysis (PPSA) has been introduced as an extension of PCA, enabling the identification of nonlinear relationships among linear shape components through polynomial regression^17^. This makes the PPSA an ideal tool for assessing the impact of WB on the variability of the foot, providing a more comprehensive understanding of foot geometry upon loading.

Therefore, this study aimed to explore and quantify morphological differences of the foot shape during WB and NWB using PPSA. We hypothesised that PPSA would yield a more comprehensive and clinically relevant interpretation of WB–NWB differences, enabling a deeper understanding of foot geometry changes in WB conditions.

## Methods

### Ethical compliance

This retrospective matched-pairs study (level III evidence) was conducted under the Declaration of Helsinki and its subsequent amendments. Ethical approval was obtained from the Institutional Review Board for both imaging modalities (WBCT: B6702022000639; MRI: EC 2019/0887), and all participants signed informed consent. Furthermore, compliance with the Strengthening the Reporting of Observational Studies in Epidemiology (STROBE) guidelines ensured complete and adequate reporting on the study design and methodology.

### Study design and subjects

**Fig. 1:**
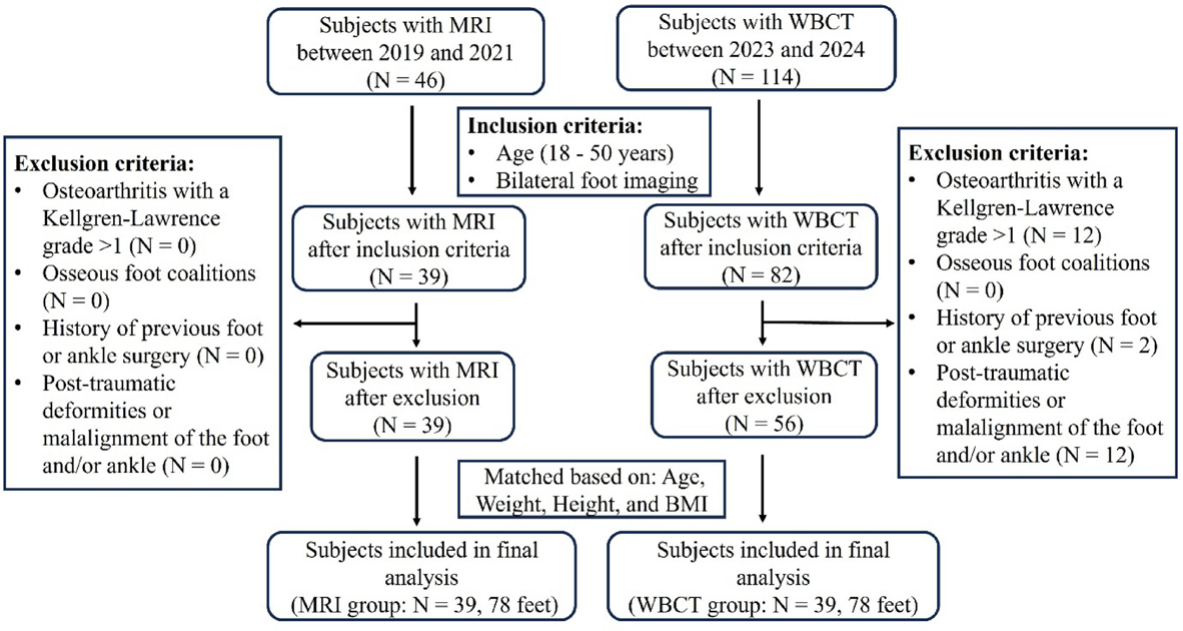
Patient selection flowchart with the inclusion and exclusion criteria.

This study used a cohort of healthy volunteers who underwent an MRI of the foot and ankle at a tertiary academic hospital between January 2021 and December 2022 for research purposes. The inclusion criteria comprised age between 18 and 50 years and bilateral foot and ankle imaging. To minimise potential confounding factors, individuals with evident lower-limb malalignment or conditions that could substantially alter load distribution were excluded from the study cohort. The exclusion criteria included osteoarthritis (Kellgren-Lawrence grade > 1), osseous foot coalitions, previous foot/ankle surgery, and post-traumatic malalignment or deformities of the foot and/or ankle region. The inclusion and exclusion criteria for these subjects were initially applied by JL, a board-certified orthopaedic surgeon. Two fellowship-trained foot and ankle surgeons (MP and AB) independently validated the patient selection, excluding inconsistent cases. Among the 46 MRI scans performed during this period, 39 subjects (mean age = 25.31 years, SD = 8.25) were confirmed to be eligible. Our WBCT database for this cohort was searched between August 2023 and July 2024 using the same inclusion and exclusion criteria. Among the 114 scans performed during this period, 39 eligible patients (mean age = 29.92, SD = 10.99) were confirmed after a matched-pairs design was applied. Although the WBCT cohort was retrieved from a clinical database, all included participants met the same inclusion and exclusion criteria as the MRI group and were clinically confirmed to have structurally normal feet. The indications for WBCT were unrelated to bony foot morphology: 24 cases involved soft-tissue complaints, 11 involved degenerative hip or knee conditions, and 4 involved non-specific ankle pain. The patient selection process is summarised in Fig. 1. A priori power analysis was not feasible due to the absence of established effect sizes for PPSA-based statistical shape analyses. Instead, sample size was determined based on comparability with prior foot SSM studies, which report sample sizes ranging from 40 to 140 feet^10,18,19^. The present study includes 78 WB feet and 78 NWB feet, placing it within—or above—the typical sample-size range used in 3D foot SSM.

A matched-pairs design was employed to pair subjects in the WB and NWB groups based on age, weight, height, and body mass index (BMI). The normality of these variables was assessed via the Shapiro–Wilk test. As variables (age, weight, height, and BMI) were not normally distributed, differences between groups were evaluated via the nonparametric Wilcoxon signed-rank test. To account for multiple comparisons, P values were adjusted via the false discovery rate (FDR) method, with an FDR-adjusted P value < 0.05 considered statistically significant. All the statistical analyses were performed in MATLAB R2022b (MathWorks, Natick, MA, USA) via the Statistics and Machine Learning Toolbox.

WBCT imaging was performed via a HiRise scanner (Curvebeam, Hatfield, PA, USA) with the following parameters: tube voltage, 130 kV; tube current, 6.5 mAs; pixel size, 0.5 mm; and slice thickness, 0.5 mm. During WBCT acquisition, participants were instructed to stand upright with a natural posture and an even weight distribution on both feet. No additional devices were used to quantify the exact load distribution between the feet. MRI scans were acquired using a 3D T1-weighted MPRAGE sequence with isotropic voxel dimensions (0.90 × 0.90 × 0.90 mm; repetition time, 2200 ms; echo time, 2.38 ms) on a 3-Tesla Magnetom Trio-Tim system (Siemens, Erlangen, Germany). During MRI acquisition, participants were positioned supine with the foot placed in a relaxed neutral position within a dedicated ankle coil. The same positioning protocol was applied to all participants to ensure consistent foot orientation and minimise positional variability during scanning.

WBCT and MRI images were exported in Digital Imaging and Communications in Medicine (DICOM) format and processed for segmentation. Initial bone segmentation was performed using automated algorithms that had been validated in prior studies for both MRI and WBCT datasets^20,21^. In previous validation studies, both MRI- and WBCT-based automated segmentation approaches have demonstrated high agreement with expert manual annotations, reporting Dice similarity coefficients around 0.95 and Hausdorff distances below 2 mm, supporting their use as reliable preprocessing steps^20,21^. In the present study, all automated segmentations were visually reviewed and manually refined when necessary by a board-certified orthopaedic surgeon (JL) to ensure anatomical accuracy. This step used Materialise’s Interactive Medical Image Control System (Mimics® v24.0, Materialise, Leuven, Belgium). The finalised 3D footbone models were reconstructed and exported in stereolithography (STL) format for subsequent analysis via dedicated open-source MATLAB toolboxes: PPSA Builder and SSM Builder.

### Registration and shape model construction

Dense anatomical point correspondence was obtained from the segmented structures by individually registering each subject’s complete foot skeleton to an isotropic template mesh comprising 34,362 vertices. The same nonrigid registration procedure was applied to meshes derived from WB and NWB acquisitions, ensuring consistent homologous landmark representation across subjects and conditions. Consequently, all bones were represented by homologous sets of dense landmarks. Subsequently, a Procrustes superimposition was applied to the whole-foot meshes to remove differences in translation and rotation, and to normalise global scale differences between samples. The scaling factor was estimated automatically during the Procrustes transformation, thereby normalising all samples to the template scale^19^. To ensure consistent anatomical orientation, the alignment was further refined using anatomical contact points corresponding to the WB tripod of the foot (most plantar points of the sesamoids (medial and lateral), the fifth metatarsal, and the calcaneus)^10^. For each subject, both right and left morphologies were available. All the left geometries were reflected into a right-sided geometry. Finally, both PCA and PPSA were applied. The PPSA captured the nonlinearity in the PCA via 2nd-order polynomial regression while maintaining 98% of the variance, following the methodology introduced by Duquesne et al.^17^. All registration and shape model constructions were performed via the MATLAB Central File Exchange^22^.

Three complementary performance metrics were employed to quantitatively evaluate the generated 3D shape models. In-model accuracy was assessed by calculating the root mean squared error (RMSE) between the reconstructed shapes and the original training set landmarks. Model compactness was quantified via cumulative explained variance, indicating the efficiency with which shape variability was captured. Finally, generalisation was evaluated using a leave-one-out cross-validation (LOOCV) framework. In each fold, the statistical shape model was rebuilt using the remaining samples, and the excluded sample was reconstructed within the learned shape space using the retained modes of variation that satisfied the predefined cumulative variance threshold. For the PCA model, this was achieved by projecting the sample onto the PCA subspace and reconstructing it using the selected principal components. For the PPSA model, the nonlinear coordinates were estimated via an optimisation procedure using the fitted PPSA manifold, after which the corresponding shape reconstruction was obtained. As all meshes share point-to-point correspondence, reconstruction is performed directly in the statistical shape space rather than via surface fitting. The RMSE between the reconstructed shape and the original sample was then calculated^10^.

### Automated measurements

**Fig. 2:**
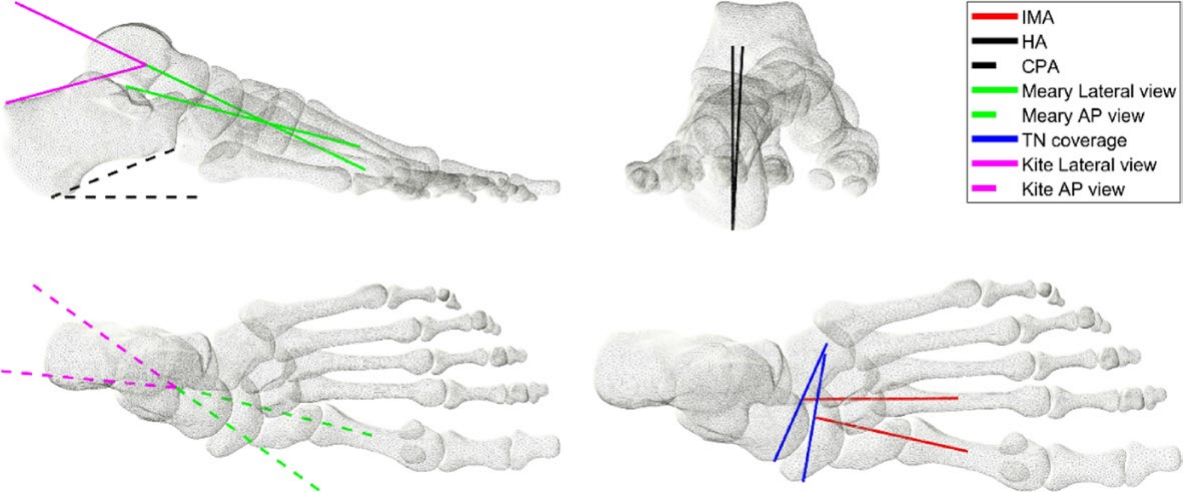
Skeletal landmark–based anatomical measurements of the foot. Automated measurements were performed on all 3D foot models to quantify anatomical alignment based on predefined skeletal landmarks. The measured parameters included the intermetatarsal angle (IMA), hindfoot alignment (HA), talonavicular coverage angle (TN), and calcaneal pitch angle (CPA). Additional anatomical reference projections illustrate Meary’s angle in both the lateral and anterior–posterior (AP) views and Kite’s angle in the lateral and AP views.

Automated measurements were performed on all the WB and NWB 3D foot models via MATLAB, following previously validated methods, including the definitions of anatomical axes and alignment angles described in Van den Borre et al.^20^. PCA was applied to fit the bone axes, which were subsequently projected onto anatomical planes to derive clinically relevant alignment angles (Fig. 2). The intermetatarsal angle (IMA) was defined as the angle between the first principal axes of the first and second metatarsals in the axial plane^20^. Meary’s angle was calculated as the angle between the talar axis and the line connecting the proximal and distal ends of the first metatarsal. It was assessed in both the sagittal and axial planes to capture deviations in longitudinal alignment^20^. The kite angle was determined as the angle between the principal axes of the talus and calcaneus, with projections independently analysed in the sagittal and axial planes to evaluate the vertical and mediolateral relationships^20^. The calcaneal pitch angle was defined as the angle between the ground plane and a line connecting the most plantar point of the calcaneus with the lowest anterior point within the anterior 10% of its surface, measured along the anteroposterior axis^20^. For the talonavicular coverage angle, linear regression was used to fit one line along the dorsal surface of the navicular head and another line along the anterior–inferior aspect of the talar head; the angle between these lines, projected onto the axial plane, was used to quantify the degree of joint uncovering^20^. Hindfoot alignment was measured as the angle in the coronal plane between the vertical axis (z-axis) and a vector extending from the most plantar calcaneal point to the centroid of the talar dome articular surface^20^. All automated measurements, including the mean ± standard deviation (SD) and range, were statistically analysed to compute descriptive statistics. Between-group comparisons were performed via independent two-sample t tests with Welch’s correction to account for unequal variances. A significance threshold of *P* < 0.05 was applied.

### Differences between the WB and NWB foot models

To analyse differences in foot geometry between the WB and NWB conditions, the mean shapes of both datasets were computed and compared, and a distance map was visualised between the mean WB and NWB foot models. Subsequently, pointwise statistical analyses using Student’s t-tests (α = 0.05) were conducted at each vertex to assess differences between the WB and NWB groups. Before these tests, the normality of the pointwise data along each axis was confirmed. The results were further validated using FDR correction to account for multiple comparisons across vertices. Statistical comparisons were conducted separately for the X (medio-lateral), Y (antero-posterior), and Z (supero-inferior) axes.

## Results

### Subject demographics

After FDR correction, the Wilcoxon signed-rank test revealed no significant differences in age, weight, height, or BMI between the WB and NWB groups (*P* > 0.05). Table 1 summarises the demographic characteristics of the study population.

**Table 1:** Demographic data of the study cohort.

	WB (WBCT-based)	NWB (MRI-based)	FDR
Age(years)	29.92 ± 10.99 (18–50)	25.31 ± 8.25 (18–50)	0.1304
Weight(Kg)	72.00 ± 13.89 (37–115)	69.15 ± 9.81 (50–89)	0.3990
Height (CM)	174.21 ± 12.58 (146–202)	175.00 ± 8.51 (156–192)	0.7448
BMI(Kg/m^2^)	23.61 ± 3.12 (17.36–32.03)	22.49 ± 2.03 (17.5–28.26)	0.1304
Side(Lift/Right)	39/39	39/39	
Sex(Male/Female)	20/19	20/19	

Values are presented as means ± SDs (ranges) or numbers.

WB: weight-bearing group; NWB: non-weight-bearing group; BMI: body mass index; FDR: false discovery rate.

### Automated measurements

Automated measurements revealed significant differences between the WB and NWB groups in multiple alignment parameters, particularly those related to forefoot abduction and joint coverage (*P* < 0.05). No significant difference was observed in the hindfoot alignment angle or the Kite angle measured in the AP view (*P* > 0.05). The detailed statistical results are presented in Table 2.

**Table 2:** Values of automated imaging measurements for the WB and NWB groups.

	WB (WBCT-based)	NWB (MRI-based)	*P* Value
Meary’s angle Lateral view	6.01 ± 4.27 (0.57 to 17.19)	11.80 ± 8.04 (1.11 to 32.74)	< 0.0001
Meary’s angle AP view	24.83 ± 9.83 (6.96 to 44.34)	20.36 ± 9.34 (6.17 to 42.92)	0.005
Kite angle lateral view	35.51 ± 4.19 (27.20 to 47.50)	28.36 ± 4.72 (17.76 to 40.90)	< 0.0001
Kite angle AP view	24.19 ± 5.42 (11.15 to 34.38)	24.40 ± 3.98 (16.08 to 32.94)	0.790
Calcaneal pitch angle	23.94 ± 4.51 (15.78 to 39.06)	29.13 ± 15.95 (6.52 to 60.19)	0.008
Hindfoot alignment angle (− = varus, + = valgus)	0.44 ± 4.87 (-8.19 to 11.55)	1.34 ± 10.69 (-14.98 to 20.22)	0.513
Talonavicular coverage angle	10.29 ± 5.11 (1.86 to 23.30)	5.05 ± 3.43 (0.58 to 20.52)	< 0.0001
Intermetatarsal angle	10.43 ± 2.04 (5.96 to 16.04)	8.10 ± 2.97 (2.40 to 15.93)	< 0.0001

Values are presented as mean ± standard deviation (range) or number.

WB: weight-bearing group; NWB: non-weight-bearing group; AP: anterior–posterior.

### Shape model evaluation

**Fig. 3:**
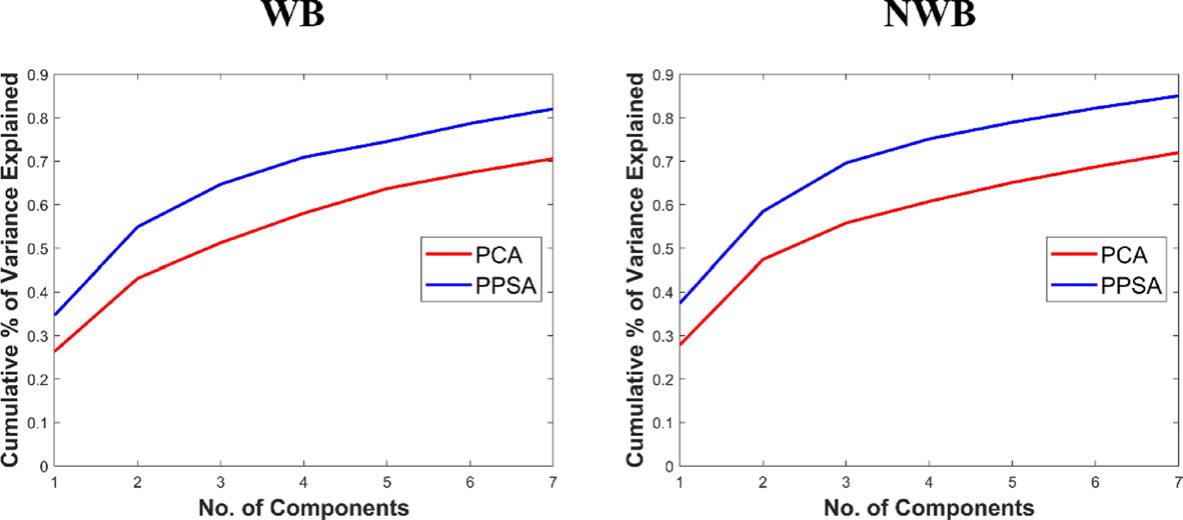
PCA and PPSA cumulative variance. The cumulative variance of the PCA (red) and PPSA (blue) analysis increases with increasing number of components for the WB (left) and NWB (right) models.

Figure 3 presents the model compactness as the cumulative explained variance of the NWB and WB models as a function of the increasing number of PCA or PPSA components. Model compactness increased to 34.6% in the WB model for the first PC when the nonlinear PPSA method was applied, as opposed to 26.3% when linear PCA was used. The in-sample RMSE of the PPSA/PCA model averaged 2.5/4.1 mm for the WB model and 3.2/5.9 mm for the NWB model, whereas the out-of-sample accuracy (generalizability) was 2.5/4.33 mm for the WB model and 3.3/6.1 mm for the NWB model. All the RMSE values were computed via shape modes sufficient to retain 80% of the cumulative explained variance in each model.

### Principal components of foot models

**Fig. 4:**
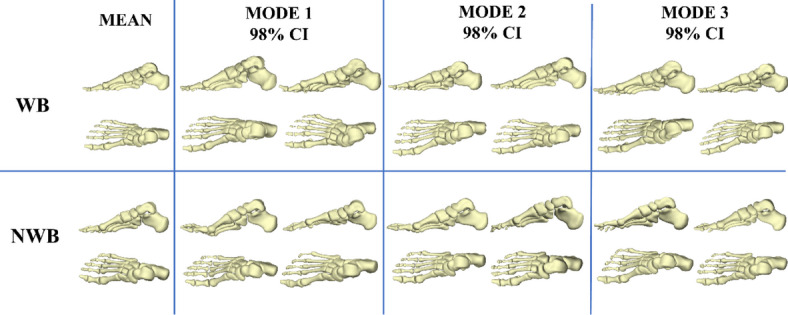
Mean shape and principal modes of variation. Medial and superior view of the mean shape for the WB (top) and NWB (bottom) of the PPSA-based models (Column 1). For both WB and NWB, the first three principal modes of variation within 98% confidence interval (CI) limits are visualised (columns 2–4).

The principal components (PCs) of foot morphology derived from PPSA are shown in Fig. 4. In the WB group, the first three PCs accounted for 64.7% of the total variance, with PC1 (34.6%) representing midfoot abduction/adduction, PC2 (20.3%) reflecting variations in medial longitudinal arch height, and PC3 (9.8%) capturing overall foot bone size. In the NWB group, the first three PCs explained 69.6% of the total variance. Specifically, PC1 (37.4%) described the phalangeal orientation, PC2 (21.0%) primarily reflected variations in the calcaneal tilt in the sagittal plane, and PC3 (11.2%) reflected hindfoot alignment (adduction/abduction).

### Mean differences between the WB and NWB groups

**Fig. 5:**
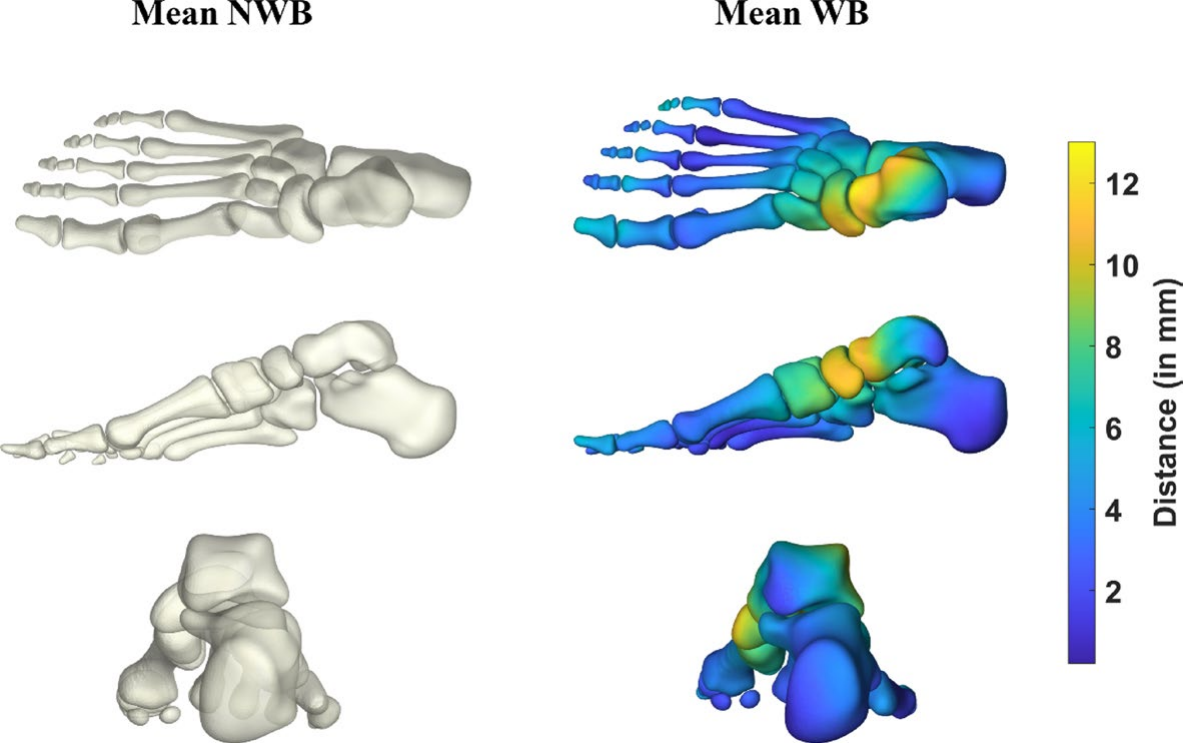
Distance map between the WB and NWB shapes. The distance plot between the mean WB and NWB shape highlights the most pronounced difference at the talonavicular level.

**Fig. 6:**
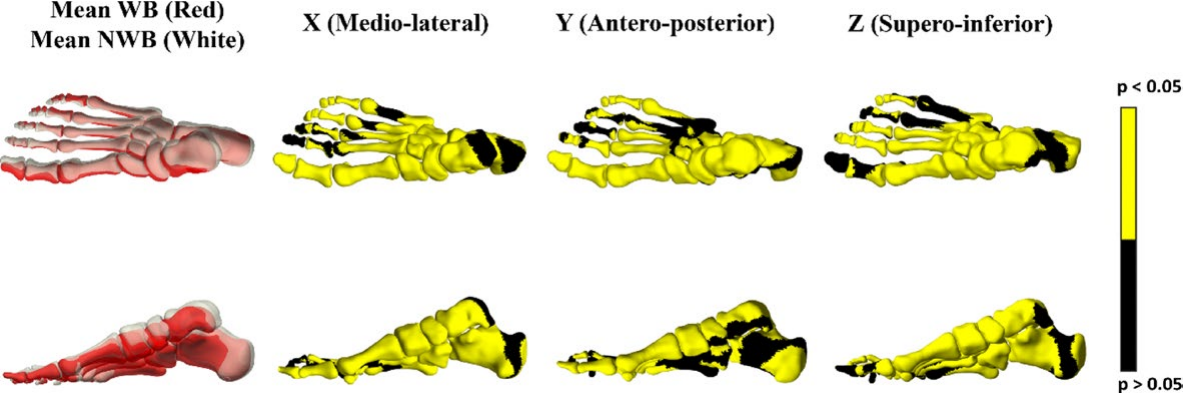
Overlay of the WB and NWB mean foot models. Overlay of the mean foot models from the WB (red) and NWB (white) groups, with pointwise statistical maps showing differences along the X (medio-lateral), Y (antero-posterior), and Z (supero-inferior) axes. Multiple viewing angles are presented for both groups. Statistically significant differences (p < 0.05, FDR-corrected) are highlighted in yellow.

Significant morphological differences were observed between the mean foot models of the WB and NWB groups. Overlaying NWB models onto WB models and visualising via distance mapping revealed deviations of up to 13 mm, predominantly around the talonavicular joint (Fig. 5). Figure 6 presents the computed pointwise differences along the three coordinate axes (X, Y, and Z) from multiple viewing perspectives for the WB and NWB groups. Regions of significant deformation are highlighted in yellow (*P* < 0.05, FDR-corrected), revealing distinct spatial patterns across all three axes.

## Discussion

This study aimed to investigate geometric alterations in foot morphology under WB conditions using PPSA, a nonlinear shape modelling technique. The principal findings of our study demonstrated significant WB-induced alterations—including forefoot abduction, midfoot flattening, and hindfoot realignment—most prominently at the talocalcaneonavicular joint.

By combining WBCT with the recently developed PPSA, this study assessed the compactness and reconstruction performance of the resulting shape models. Compared with PCA, PPSA demonstrated improved model compactness (Fig. 3) and lower in-sample reconstruction error. Out-of-sample accuracy was comparable between PPSA and PCA, indicating that this increased compactness does not come at the expense of generalizability. With this modelling approach, we further mapped detailed morphological variations of the foot under WB conditions (Fig. 4). In addition to the talonavicular joint, other notable morphological alterations included forefoot abduction, dorsiflexion of the metatarsals and phalanges, flattening of the medial longitudinal arch, internal rotation of the talus, and calcaneal valgus. This aligns with previous studies that recognised the talocalcaneonavicular joint—or coxa pedis—as crucial for stabilising the longitudinal arch and resisting the downwards force exerted by the talar head during WB^23^.

In the geometric changes of the forefoot, WB conditions induce abduction of the phalanges and metatarsals in the transverse plane and dorsiflexion in the sagittal plane. Pointwise surface distance mapping and statistical shape comparisons supported these findings by revealing clear local displacement patterns between the WB and NWB conditions (Figs. 5, 6). Correspondingly, the IMA significantly increased under WB conditions (P < 0.05), reflecting load-dependent widening and splaying of the forefoot during axial loading (Table 2). These results corroborate previous work by Broos et al., who reported that WBCT-derived parameters (e.g., cuboid height, calcaneal pitch, talo-calcaneal angle, Meary’s angle, and IMA) significantly differ from NWB radiographic measurements^24^. Clinically, axial loading leads to forefoot widening, most notably reflected by a marked increase in the IMA—a change often underestimated on NWB imaging modalities^25^. Unlike standard radiographs, which are limited by projectional distortion and structural superimposition, WBCT allows precise quantification of the IMA, metatarsal rotation, and pronation^25^. These parameters are critical in managing hallux valgus and other forefoot deformities, directly informing the choice of surgical technique and the extent of correction needed. For example, increased metatarsal pronation may warrant a rotational osteotomy or a triplanar Lapidus procedure to achieve adequate multiplanar correction^26^.

WBCT demonstrated the medial longitudinal arch flattening under WB conditions in the midfoot. Shape and distance colour mapping illustrated pronounced displacement, particularly around the talocalcaneonavicular joint (Figs. 5, 6). Meary’s angle in lateral and AP views demonstrated significant alterations, indicating load-dependent changes in midfoot sagittal and transverse alignment during axial loading. Similarly, the talonavicular coverage angle increased significantly under WB conditions, reflecting functional adaptation of the midfoot joint complex, as illustrated in Table 2. These movements can be attributed to plantar flexion and abduction of the midfoot bones, which reduce the lateral tarsometatarsal and calcaneal pitch angles^27^. Several studies have similarly reported an increase in the arch index under loading, indicative of reduced arch height, across various foot types, including cavus, neutral, and planus^28^. From a clinical perspective, midfoot deformation under WB is not merely structural but also essential for shock absorption and postural balance during gait. Accurate assessment is crucial when this adaptive mechanism becomes pathological, such as cuneometatarsal joint instability, early-stage osteoarthritis, and subtle joint subluxations, as in cases of instability or progressive foot collapse^29,30^.

WBCT imaging of the hindfoot revealed distinct morphological adaptations of the subtalar joint under axial loading. Shape and distance colour mapping support these findings, showing the greatest morphological variability in the talar head, talar neck, and subtalar joint facets, as illustrated (Figs. 5, 6). WB induces calcaneal valgus and internal rotation of the talus relative to the NWB state, contributing to load-dependent realignment of the hindfoot^24,25^. Among the hindfoot parameters, the lateral Kite angle increased significantly under WB conditions, reflecting subtalar joint repositioning during load-bearing. The calcaneal pitch angle decreased significantly under WB conditions, as shown in Table 2, indicating that medial arch flattening and posterior segment adjustment occurred in response to axial loading^31^. These findings align with physiological hindfoot realignment during standing and challenge the traditional view of the talus and calcaneus as rigid units, highlighting the critical role of the subtalar joint in multiplanar adaptation^32^. WBCT uniquely captures these load-induced and rotational subtalar mechanics frequently overlooked in NWB imaging^33^.

It is important to note that these load-dependent morphological adaptations were observed in a cohort of structurally normal, healthy feet. Because individuals with deformities, osteoarthritis, prior trauma, or other pathological conditions were excluded, the WB–NWB differences described above may not be generalizable to pathological feet. In addition, although participants were instructed to distribute their weight evenly during WBCT acquisition, minor variations in load distribution between the two feet cannot be excluded. However, this reflects standard clinical WBCT practice and is not expected to introduce systematic bias in the observed group-level morphological differences^3,6,10^.

Although conventional radiographic parameters such as the AP Kite angle and hindfoot alignment angle did not significantly differ between the WB and NWB conditions in this study, this does not imply an absence of morphological changes in the hindfoot. The lack of statistical significance might be attributed to the intrinsic structural stability and soft tissue resilience characteristic of younger healthy individuals (study cohort aged 18–50). These results highlight the limitations of angle-based measurements in capturing complex, multidirectional shape deformations. As shown in Fig. 6, geometric morphometric analysis revealed statistically significant pointwise displacements across all three spatial axes—particularly in the subtalar joint, calcaneus, and talus. This underscores subtle yet systematic morphological adaptations that are not fully captured by conventional angular metrics.

Building on conventional radiographic parameters and geometric morphometric analyses, the present findings underscore the importance of assessing foot morphology under physiologically relevant loading conditions and from a unified, whole-foot perspective. The foot operates as an integrated kinetic chain in which the forefoot, midfoot, and hindfoot undergo coupled, multiplanar motions that collectively contribute to arch stability, load distribution, and joint alignment^33^. WB imaging captures these coordinated adaptations across all foot regions, thereby revealing load-dependent compensatory mechanisms that remain undetectable in NWB conditions^11^.

The clinical relevance of these load-dependent morphological adaptations is evident in several common scenarios. In Lisfranc injuries, NWB CT or MRI may appear to show isolated tarsometatarsal joint involvement, whereas WBCT often reveals additional load-dependent instabilities in adjacent midfoot articulations—such as intercuneiform or cuneonavicular malalignment—that can influence decisions regarding immobilisation or surgical fixation^34,35^. Likewise, in hindfoot valgus or varus deformity, NWB imaging may suggest isolated calcaneal malalignment, whereas WBCT frequently reveals increased hindfoot valgus or varus, associated forefoot widening, metatarsal pronation, or midfoot collapse—information essential for selecting combined or multiplanar corrective procedures^25^.

These examples underscore why establishing normative WB–NWB morphological baselines is important: they provide a reference for identifying pathological, load-dependent deviations. Within this context, PPSA-derived 3D shape metrics can complement conventional angular measures by capturing subtle, multiplanar, and cross-regional variations that are not visible on 2D imaging.

## Limitations

This study has several limitations. First, the matched-pairs study design used a cohort that underwent MRI scans and a cohort that underwent WBCT imaging. Despite this difference in imaging modality, previous research has validated the feasibility of MRI-based bone segmentation, demonstrating comparable accuracy to CT-based methods with no significant differences in segmentation outcomes^36,37^. Regarding the differences in cohorts, our centre’s experience in WBCT imaging ensured a large sample size in our database that supported adequate paired matching. Second, minor geometric discrepancies may have occurred during the segmentation process due to image noise, foot positioning variability, or registration inaccuracies. Although such factors could impose a variation within the 3D models, prior reports indicate that these differences typically remain within 0.11 mm, implying a minor interference with the obtained measurements^38^. Third, shape modelling techniques require large datasets. While the current study utilised demographically matched WB and NWB groups of 78 feet each, larger sample sizes would increase the statistical power and robustness. Fourth, both WB and NWB imaging modalities used in this study are inherently static. Although WBCT captures physiologically relevant loading conditions, neither WBCT nor MRI provides information on dynamic foot function, such as gait-dependent joint kinematics, muscle activation, or time-dependent loading patterns. As a result, the WB–NWB morphological differences reported here reflect static postures rather than functional movement. Future studies integrating WBCT-based morphometrics with dynamic assessments—such as gait analysis or fluoroscopic motion capture—may further improve understanding of load-dependent foot biomechanics.

Future research should pursue four key directions. First, increasing sample sizes and developing disease-specific reference databases—including cohorts with pathological foot conditions—to enhance diagnostic resolution and improve the statistical robustness of shape modelling. Second, exploring load-dependent postural variations that can be assessed within WBCT systems, such as single-leg stance or altered load distribution, which may provide further insight into WB adaptations of foot morphology. Third, in the longer term, integrating WBCT-derived morphometrics with dynamic assessments—such as gait analysis, fluoroscopic motion capture, or finite-element simulations—could provide complementary insights into functional foot biomechanics and potential pathways of deformity progression. Finally, evaluating how advanced morphological descriptors derived from PPSA could be incorporated into clinical workflows—pending rigorous validation in pathological cohorts—to support patient-specific assessment or device design. While these translational developments extend beyond the scope of the present study, they represent important avenues for future investigation.

## Conclusions

In conclusion, this study presents the first comprehensive application of the PPSA combined with WBCT to evaluate the geometric differences between the foot’s WB and NWB. PPSA revealed significant WB-induced alterations—including forefoot abduction, midfoot flattening, and hindfoot realignment—most prominently at the talonavicular joint. These findings underscore the value of WBCT for capturing physiologically relevant foot morphology and establish a morphological reference framework that can inform future investigations of pathological deformities and their potential clinical implications.

## Data Availability

The datasets generated and/or analysed during the current study are available from the corresponding author upon reasonable request.
